# Norisoprenoids, Sesquiterpenes and Terpenoids Content of Valpolicella Wines During Aging: Investigating Aroma Potential in Relationship to Evolution of Tobacco and Balsamic Aroma in Aged Wine

**DOI:** 10.3389/fchem.2018.00066

**Published:** 2018-03-19

**Authors:** Davide Slaghenaufi, Maurizio Ugliano

**Affiliations:** Department of Biotechnology, University of Verona, Verona, Italy

**Keywords:** wine aging, tobacco aroma, balsamic aroma, terpenoids, 1,8-cineole, 1,4-cineole

## Abstract

During wine aging, tobacco and balsamic aroma notes appear. In this paper, volatile compounds directly or potentially related to those aromas have been investigated in Corvina and Corvinone wines during aging. Corvina and Corvinone are two northern-Italy autochthonous red grape varieties, used to produce Valpolicella Classico and Amarone wines, both characterized by tobacco and balsamic aroma notes. Wines were analyzed shortly after bottling or following model aging at 60°C for 48, 72, and 168 h. Volatile compounds were analyzed by HS-SPME-GC-MS. Results showed that compounds related to tobacco aroma [β-damascenone, 3-oxo-α-ionol, (E)-1-(2,3,6-Trimethylphenyl)-buta-1,3-diene (TPB), and megastigmatrienones] increased in relationship to storage time with different patterns. β-Damascenone and 3-oxo-α-ionol rapidly increased to reach a plateau in the first 48–72 h of model aging. Instead, TPB and megastigmatrienones concentration showed a linear correlation with aging time. During model aging, several cyclic terpenes tended to increase. Among them 1,8-cineole and 1,4-cineole, previously reported to contribute to red wine eucalyptus notes increased proportionally to storage time, and this behavior was clearly associated with reactions involving α-terpineol, limonene, and terpinolene, as confirmed by studies with model wine solutions. Among other relevant volatile compounds, sesquiterpenes appear to contribute potentially balsamic and spicy aroma notes. In this study, linear sesquiterpenes (nerolidol, farnesol) underwent acid hydrolysis during long wine aging, while cyclic sesquiterpenes seemed to increase with time. The chemical pathways associated with evolution of some of the compounds investigated have been studied in model wine.

## Introduction

Valpolicella is a region located in the north-east of Italy, where the red wines “Valpolicella Classico,” Recioto and the most famous Amarone are produced. The grape varieties used to make these wines are mainly Corvina and Corvinone. Aging of the wine in cellar is common in this area, and he DOC and DOCG regulations envisage a minimum aging period before commercialization, which can last up to a minimum of 2 years in the case of Amarone, and 4 years for the Amarone Riserva. The maturation is generally carried out in big oak casks, in order to limit the contribution of wood to wine aroma.

The chemical composition of wines made by Corvina or Corvinone grapes has been little investigated. Most of these works concerned the withering process from different compositional point of view (Zamboni et al., 2008; Rolle et al., 2013; Zoccatelli et al., 2013), or about grape and wine microbiome (Lorenzini et al., 2013; Stefanini et al., 2017), while only few papers described Corvina and Corvinone wines aroma composition (Fedrizzi et al., 2011a,b; Bellincontro et al., 2016).

The olfactory descriptors often used for wines made by Corvina and Corvinone grapes, are cherry, pepper, spicy, tobacco, and balsamic (Bellincontro et al., 2016). Tobacco and balsamic aroma are also commonly associated to aged wines (McKay et al., 2010; Moio, 2016), but their chemical origin has been only marginally investigated so far. Recently megastigmatrienone (Arbulu et al., 2013; Slaghenaufi et al., 2013, 2014) and (E)-1-(2,3,6-trimethylphenyl)buta-1,3-diene (TPB) (Janusz et al., 2003) have been identified as possible contributor to tobacco aroma in wine. Likewise, recent evidences suggest that the cyclic terpenes 1,4-cineole and 1,8-cineole could contribute to balsamic aromas, but their origin is still debated (Fariña et al., 2005; Capone et al., 2011; Antalick et al., 2015).

During aging, wine aroma is modified by various reactions depending on wine composition, SO_2_ and oxygen exposure levels, pH, temperature, conditions, and time of storage. The acidic environment plays a major role on the evolution of wine bouquet, promoting a variety of chemical reactions including ester hydrolysis and formation (Díaz-Maroto et al., 2005; Makhotkina and Kilmartin, 2012; Antalick et al., 2014), oxidation, and cyclization. Hydrolysis of non-volatile glycosidic precursors as well as chemical rearrangements of certain volatile compounds arising from grapes or fermentation are thought to be two key phenomena contributing to formation of aged wine aroma (Winterhalter, 1991; Winterhalter and Skouroumounis, 1997; Skouroumounis and Sefton, 2000; Gunata, 2003). Carbocation intermediates are formed during these reactions, resulting in a variety of different reaction products with variable aroma characteristics (Wedler et al., 2015). Aroma compounds such as norisoprenoids, monoterpenes, and sesquiterpenes are associated with these reactions and are thought to be potential contributors to tobacco and balsamic aroma notes.

The aim of this work was to investigate the evolution of Valpolicella wine volatile compounds during aging, focusing on compounds potentially involved in tobacco and balsamic odor notes. Wines have been analyzed at the end of alcoholic fermentation and at different time of accelerated aging. The formation pathway of as the potent odorants 1,4- and 1,8-cineole as well as other cyclic terpenes was also investigated by means of model wine solution experiments.

## Materials and methods

### Reagents and materials

Octan-2-ol (97%), linalool (97%), terpinen-4-ol (≥95%), α-terpineol (90%), p-cymene (99%), nerol (≥97%), geraniol (98%), 1,8-cineole (99%), 1,4-cineole(≥98.5%), limonene (97%), terpinolene (≥85%), linalool oxide (≥97%), β-citronellol (95%), nerolidol (98%, mix of two isomers), farnesol (95%), bisabolol (≥93%), β-damascenone (≥98%), p-menthane-1,8-diol (≥98%), sodium chloride (≥99.5%) were supplied by Sigma Aldrich (Milan, Italy). Megastigmatrienone was from Symrise AG (Holzminden Germany) as a mix of isomers (purity 78%). Ethanol (≥98%) was furnished by Honeywell (Seelze, Germany). Tartaric acid (≥99%) was purchased from J.T. Baker (Deventer, Holland). Synthetic wine was prepared at 12% of ethanol, 3 g/L of tartaric acid, pH was adjusted to pH 3.1 with NaOH (0.5 M), studied compounds (α-terpineol, terpinen-4-ol, linalool, 1,8-cineole, limonene, p-menthane-1,8-diol, terpinolene) were individually added at 400 μg L^−1^.

### Wine samples and model aging

Wine samples used during the experiment were obtained from the vinification of Corvina and Corvinone grapes from seven different vineyard locations in the Valpolicella area. Five Corvina and two Corvinone batches were used, and all winemaking was carried out using the same vinification protocol.

Model aging was made by placing 115 mL of wine in glass vial and crimped. The crimped cap was further sealed with Araldite glue to prevent any oxygen transfer. A headspace of 0.8 mL was left corresponding to an oxygen supply of 2 mg L^−1^, which is commonly found in commercial wine bottling (Ugliano et al., 2011). Sample vials were then placed at 60°C (±0.2°C) for 48, 72, and 168 h, similar to the model aging protocol proposed by Silva Ferreira et al. (2003).

### SPME-GC-MS analysis

Volatiles have been quantified using a method previously published for the analysis of cyclic terpenes (Antalick et al., 2015; Picard et al., 2016), which was further adapted to the analysis of the other terpenoids of this study by extending extraction time. An aliquot of 5 mL of wine was placed into a 20 mL vial together with 5 mL of mQ water (18.2 MΩ-cm) and 3 g of NaCl. Five microliters of internal standard solution (octen-2-ol at 420 mg L^−1^ in ethanol) were added to the vial.

Sample was equilibrated for 1 min at 40°C. Subsequently SPME extraction was performed using a 50/30 μm divinylbenzene–carboxen–polydimethylsiloxane (DVB/CAR/PDMS) fiber (Supelco, Bellafonte, PA, USA) exposed to sample headspace for 60 min at 40°C. Desorption was done into the injector port at 250°C for 5 min in splitless mode.

GC-MS analysis was performed on an HP 7890A (Agilent Technologies, USA) gas chromatograph coupled to a 5977B quadrupole mass spectrometer, equipped with a Gerstel MPS3 autosampler (Müllheim/Ruhr, Germany). Separation was performed using a DB-WAX capillary column (30 m × 0.25, 0.25 μm film thickness, Agilent Technology, USA) and helium as carrier gas at 1.2 mL/min of constant flow rate. GC oven was programmed as follow: started at 40°C for 3 min, raised to 230°C at 4°C/min and maintained for 20 min. Mass spectrometer operated in electron ionization (EI) at 70 eV with ion source temperature at 250°C and quadrupole temperature at 150°C. For quantification, mass spectra were acquired in Selected Ion Monitoring (SIM) mode (Table 1).

**Table 1:** Retention times, retention indices, quantification ions of studied compounds.

**Compounds**	**RT (min)**	**LRI^a^ (DB-WAX)**	**Quantification ion *m/z***	**Qualifier ions *m/z***	**Identification^b^**	**LOD (μg L^−1^)**	**LOQ (μg L^−1^)**	**Repeatability (RSD %) *n* = 5**
1,4-Cineole	12.1	1,186	154	139, 125, 111	RS	0.003	0.010	5.3
Limonene	12.5	1,198	136	121, 93	RS	0.03	0.10	10.1
1,8-Cineole	13.2	1,217	154	139, 111, 108	RS	0.003	0.011	9.0
γ-Terpinene^c^	13.6	1,228	93	136, 121	LRI MS	–	–	3.6
p-Cymene	15.1	1,271	119	134, 91	RS	0.08	0.24	7.3
Terpinolene	15.6	1,283	121	136, 93	RS	0.04	0.15	5.9
cis-Linalooloxide	21.2	1,437	59	111, 94	RS	0.02	0.07	2.8
trans-Linalooloxide	22.1	1,469	59	111, 94	RS	0.02	0.07	6.8
Vitispirane 1^c^	23.6	1,523	192	177, 93	LRI MS	–	–	5.2
Vitispirane 2^c^	23.6	1,524	192	177.93	LRI MS	–	–	6.1
Linalool	24.2	1,547	71	121, 93	RS	0.08	0.25	2.2
Terpinen-1-ol	25.1	1,581	136	121, 81	LRI MS	–	–	5.8
Terpinen-4-ol	26.0	1,614	71	111, 93, 86	RS	0.02	0.05	2.7
Sesquiterpene 5^c^	28.4	1,696	204	189, 161	LRI MS	–	–	12.3
α-Terpineol	28.5	1,701	136	121, 93, 59	RS	0.23	0.70	3.6
Sesquiterpene 7^c^	29.3	1,728	204	136, 121	LRI MS	–	–	11.8
TDN ^c^	29.7	1,745	157	172, 142	LRI MS	–	–	5.8
β-Citronellol	30.4	1,771	69	82, 81, 67	RS	0.07	0.21	2.3
Nerol	31.5	1,812	93	121, 84, 69	RS	0.2	0.6	8.9
β-Damascenone	31.8	1,825	69	190, 121, 105	RS	0.01	0.03	7.5
TPB ^c^	31.9	1,828	172	157, 142	LRI MS	–	–	15.7
Geraniol	32.7	1,860	93	123, 121, 69	RS	0.06	0.2	4.8
Nerolidol 1	36.8	2,024	69	161, 136, 93	RS	0.015	0.05	2.5
Nerolidol 2	37.3	2,045	69	161, 136, 93	RS	0.015	0.05	2.7
p-Menthane-1,8-diol	38.8	2,120	81	139, 96, 59	RS	350	1,050	3.3
Megastigmatrienone 1	40.1	2,154	190	175, 147, 133	RS	0.13	0.40	8.3
Megastigmatrienone 2	41.1	2,196	190	175, 147, 133	RS	0.37	1.17	9.9
Bisabolol	41.3	2,206	204	119, 109	RS	0.03	0.10	8.1
Megastigmatrienone 3	42.6	2,255	190	175, 147, 133	RS	0.05	0.14	5.7
Megastigmatrienone 4	43.3	2,286	190	175, 147, 133	RS	0.18	0.5	7.1
8-Hydroxylinalool^c^	43.5	2,292	71	137, 67	LRI MS	–	–	14.4
Farnesol 1	43.7	2,300	69	136, 93, 81	RS	0.03	0.10	4.5
Farnesol 2	44.4	2,327	69	136, 93, 81	RS	0.03	0.10	7.1
3-Oxo-α-ionol^c^	50.1	2,555	108	152	LRI MS	–	–	4.9

a *Linear Retention Index (LRI) were determined on DB-WAX polar column, as described by van Den Dool and Kratz (1963)*.

b *RS identified using reference standard; LRI MS tentatively identified by comparing the Linear Retention Index and mass spectra with those of literature*.

c *Reference standard not available, LOD and LOQ couldn't be determined*.

### Statistical analysis

GC-MS data were submitted to multivariate statistical analysis using Unscrambler ®X 10.4 (CAMO Software, Oslo, Norway). Data were submitted to Pareto scaling prior to unsupervised principal component analysis (PCA), that was used to identify the compounds more correlated with wine aging.

## Results and discussion

Wine contains several dozens of volatile compounds potentially contributing to its aroma properties (Francis and Newton, 2005). Aging can deeply affect their concentration through various chemical reactions, so that wine aroma characteristics can be deeply modified after a period of storage. The impact of aging on esters, acids, and fusel alcohols, all contributing to the fruity and vinous character of wine, have been extensively studied (Díaz-Maroto et al., 2005; Makhotkina and Kilmartin, 2012; Antalick et al., 2014). On the contrary, the chemical basis of the evolution of balsamic or tobacco notes during aging has not been systematically explored to date, in spite of the fact that expression of these aroma attributes is often associated with wine storage (McKay et al., 2010; Moio, 2016).

A preliminary analysis of the literature was carried out in order to identify previously reported wine aroma components that could potentially contribute to these attributes (Table 2). A set of 34 volatile compounds was developed (Table 1) and these were then quantified in the seven wines by HS-SPME-GC-MS, after model aging by heating wines at 60°C for 48, 72, and 168 h.

**Table 2:** Aroma descriptors and occurrence in wine of potential contributors to balsamic and tobacco odor.

**Compounds**	**Odor note**	**Presence**
1,4-Cineole	Hay, eucalyptol like, camphoraceous (Antalick et al., 2015)	Antalick et al., 2015
1,8-Cineole	Eucalyptus, camphoraceous, cool (Fariña et al., 2005)	Hervé et al., 2003; Fariña et al., 2005
Terpinolene	Lime, pine, turpentine (Gocmen et al., 2004)	Dziadas and Jelen, 2010
Vitispirane 1	Camphor (Genovese et al., 2007)	Simpson et al., 1977
Vitispirane 2	Camphor (Genovese et al., 2007)	Simpson et al., 1977
α-Terpineol	Piney (Tatum et al., 1975)	Schreier and Drawert, 1974; Rapp et al., 1985
β-Damascenone	Fruity, balsamic, tobacco (Weyerstahl et al., 1991)	Schreier and Drawert, 1974
(E)-1-(2,3,6-trimethylphenyl)buta-1,3-diene (TPB)	Tobacco, floral, geranium (Janusz et al., 2003)	Janusz et al., 2003; Cox et al., 2005
Megastigmatrienone 1	Wood, tobacco (Weyerstahl et al., 1991; Slaghenaufi et al., 2014)	Slaghenaufi et al., 2014
Megastigmatrienone 2	Smoky, tobacco (Weyerstahl et al., 1991; Slaghenaufi et al., 2014)	Slaghenaufi et al., 2014
Megastigmatrienone 3	Burnt (Weyerstahl et al., 1991; Slaghenaufi et al., 2014)	Slaghenaufi et al., 2014
Megastigmatrienone 4	Burnt, wood (Weyerstahl et al., 1991; Slaghenaufi et al., 2014)	Slaghenaufi et al., 2014
3-Oxo-α-ionol	Tobacco (Ribéreau-Gayon et al., 2006)	Strauss et al., 1987

**Figure 1:**
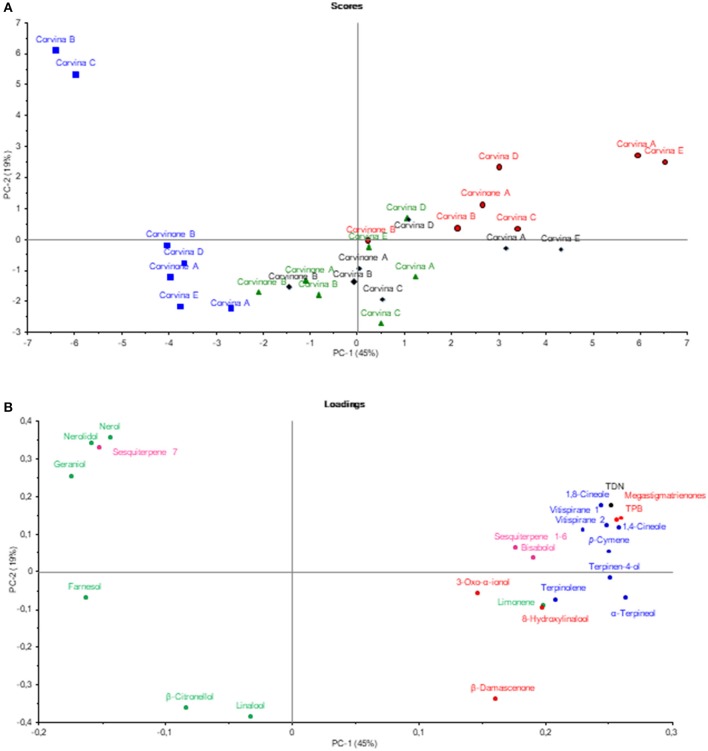
Principal component analysis showing samples scores **(A)**, and correlation loadings **(B)**. Samples (scores) are labeled with their aging time (min): no aged (blue), 48 min (green), 72 min (black), 168 min (red). Loadings have been colored according to their aroma descriptor class: balsamic (blue), tobacco (red), fruity-flowery (green), petroleum (black), unknown (pink).

In a first step, the GC-MS data related to the target aroma compounds as well as to their possible precursors and/or derivatives were analyzed by Principal Component Analysis (PCA) (Figure 1) in order to have a global view on the aging phenomenon. It emerged that samples were separated on the first component (45%) according to the times of aging, only samples aged for 48 and 72 h were overlapped. The compounds that mostly contributed to the separation on the first dimension were megastigmatrienone, TDN, TPB, 1,8-cineole, 1,4-cineole, α-terpineol, terpinen-4-ol, and vitispirane, all clearly associate with aging time. Conversely, farnesol nerolidol, nerol, geraniol, linalool, and β-citronellol seemed to characterize young wines before aging. The group of young wine was further divided into two groups, separated along PC 2: one characterized by the nerolidol, nerol, geraniol content, the other linked to the amount of linalool and β-citronellol.

### Tobacco-like aroma compounds

Our literature analysis indicated that the compounds β-damascenone (Weyerstahl et al., 1991), megastigmatrienone (Slaghenaufi et al., 2014), 3-oxo-α-ionol (Ribéreau-Gayon et al., 2006), and TPB (Janusz et al., 2003) as possible contributors to tobacco-like aromas.

**Figure 2:**
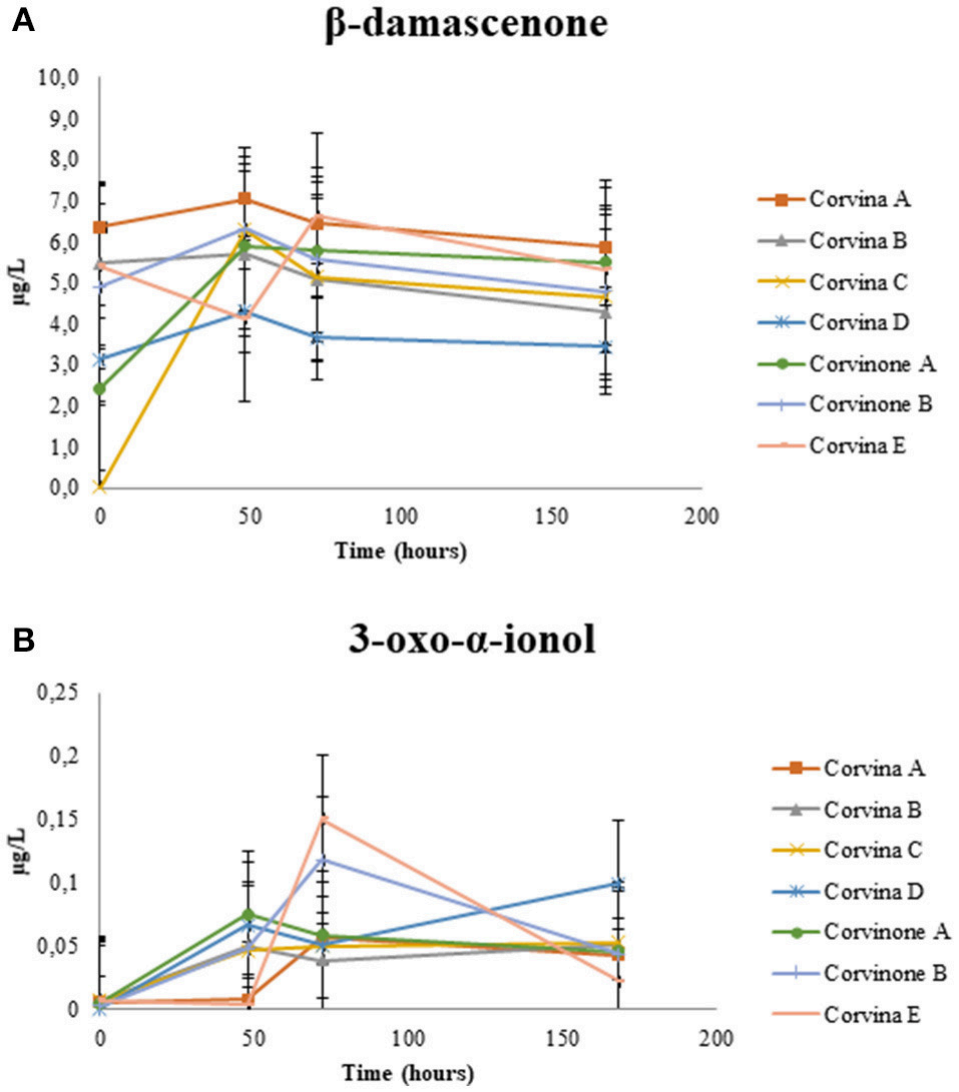
Evolution of β-damascenone **(A)** and 3-oxo-α-ionol **(B)** during wine aging.

**Figure 3:**
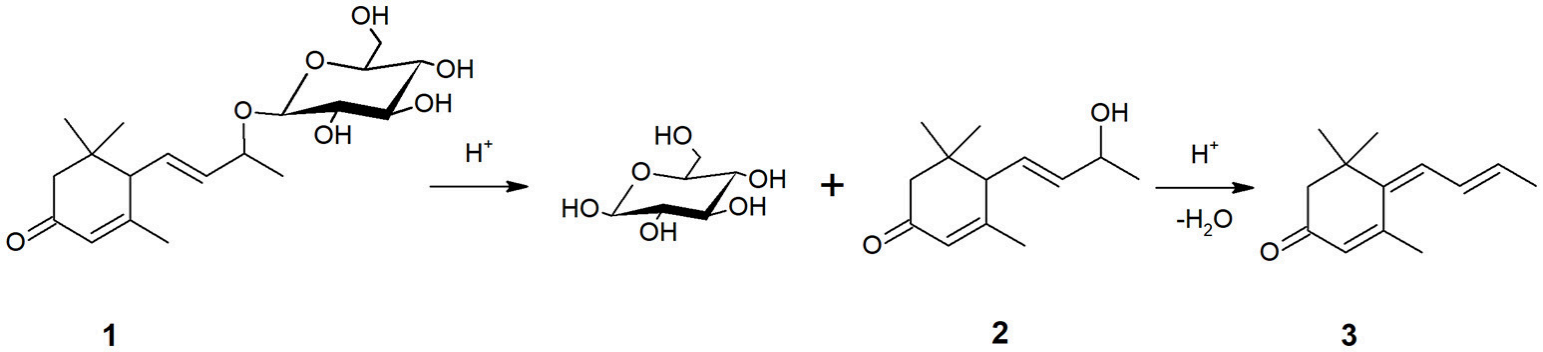
Formation of megastigmatrienone (**3**) from 3-oxo-α-ionol (**2**) and its glucoside precursor (**1**) during aging.

The data related to the evolution of β-damascenone are shown in Figure 2. An initial increase was observed, although its concentration eventually dropped at levels lower than those present at the beginning of aging, as observed also by Loscos et al. (2010). A similar behavior during model aging was observed for 3-oxo-α-ionol (Figure 3). Different precursors of both compounds have been identified in wine and grapes (Baumes et al., 1994) either as glycoconjugates and free norisoprenoids (Skouroumounis et al., 1992). Acid hydrolysis of glycosidic precursors and subsequent possible rearrangements are likely to be the reason for the initial increase observed for these two compounds. In the case of 3-oxo-α-ionol, this initial increase is probably due to hydrolysis of its glycosidic precursors followed by molecular rearrangements eventually leading to megastigmatrienone, as it can be seen in Figure 3 (Slaghenaufi et al., 2014).

**Figure 4:**
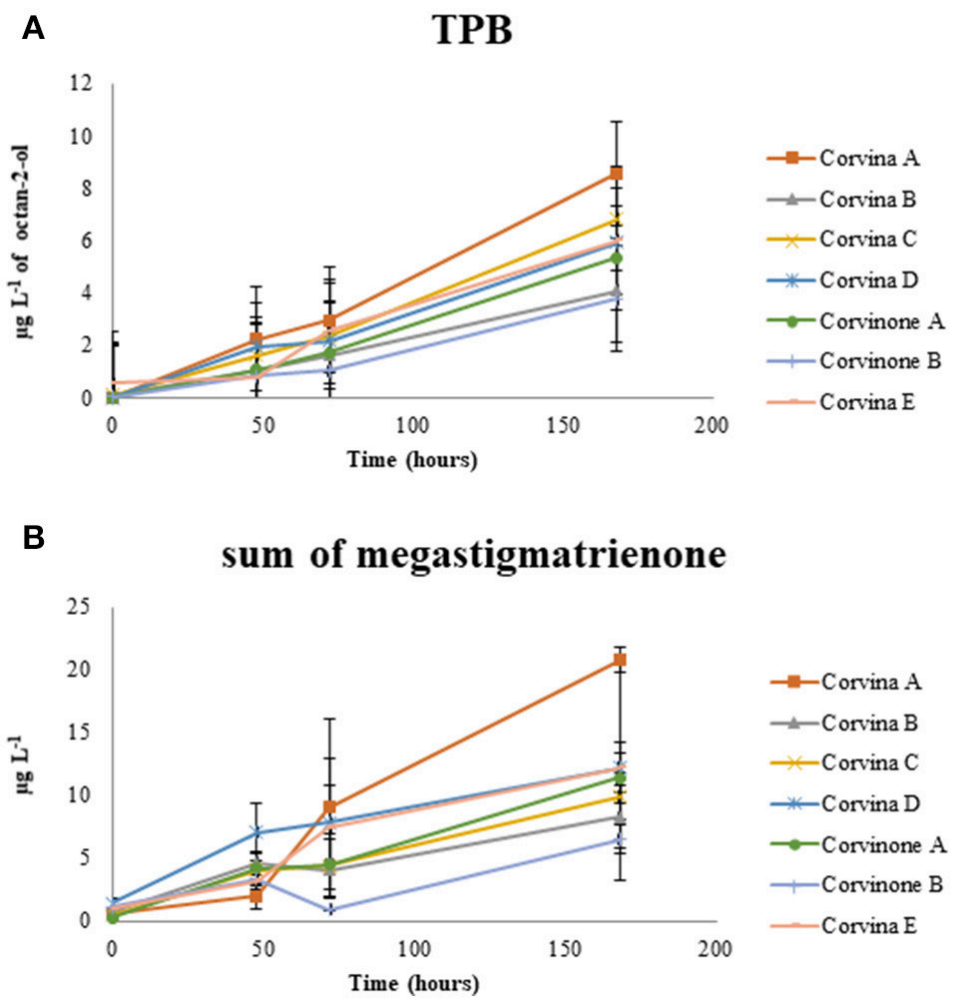
Evolution of the tobacco odor zone associated to TPB **(A)** and megastigmatrienone (sum of isomers) **(B)** during aging.

This latter process is of particular interest because megastigmatrienones have been indicated as important contributors to tobacco aroma attributes in wines and spirits (Slaghenaufi et al., 2016). The evolution of the sum of the four isomers of megastigmatrienones measured is shown in Figure 4, indicating that these compounds accumulate progressively during wine storage (*R*^2^ = 0.696), and should be probably considered as end products of 3-oxo-α-ionol degradation. Previous data showed that aging of wine in contact with oak can increase wine megastigatrienone content (Slaghenaufi et al., 2014). Given that the wines analyzed in this study have not been in contact with oak wood, our data indicate that megastigmatrienones can be generated from precursors directly derived from grape, as proposed by Slaghenaufi et al. (2014).

**Figure 5:**
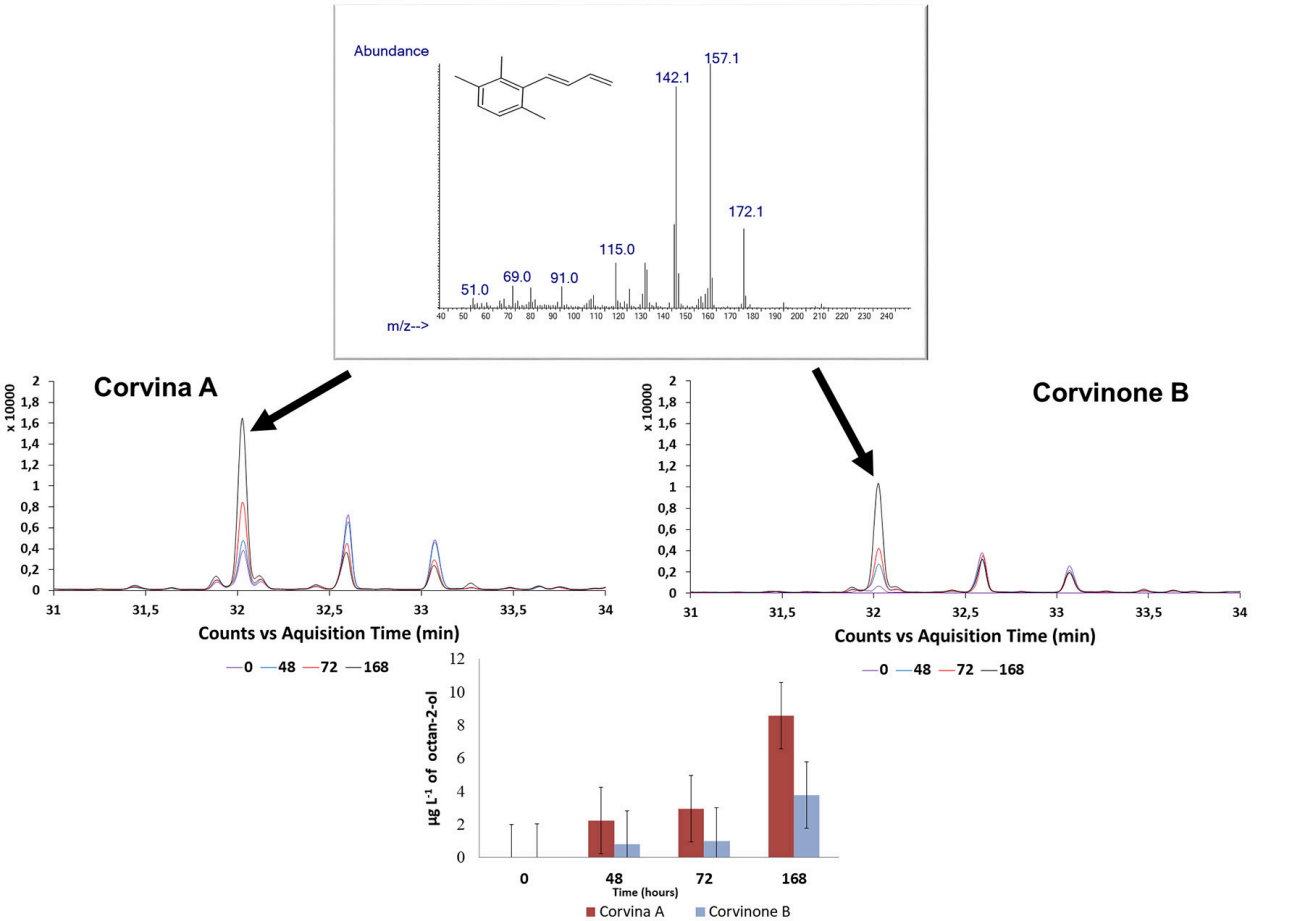
Extracted ion chromatograms (*m/z:* 172) and mass spectra of the peak (32 min) at the 4 levels of aging, tentatively identified as TPB. Samples: Corvina A, Corvinone B.

During preliminary GC-O-MS assessment of samples from model aging, an odor zone described as “tobacco” with a Linear Retention Index (LRI) of 1828 was observed (Figure 5; Table 1). The mass spectrum of the peak associated with this odor region was similar to that reported for TPB, with odor description and LRI also matching those reported in the literature (Janusz et al., 2003). Similar to megastigmatrienone, evolution of this peak during aging showed that this compound accumulated progressively with aging (*R*^2^ = 0.849; Figure 4), with a trend that was well correlated with that of megastigmatrienones (*R*^2^ = 0.696).

All the compounds related to tobacco aroma generally increase or stay stable during wine aging. These results provided a chemical point of view to the empirical observation that tobacco aroma grows up with time, most due to the accumulation of volatile compounds such as megastigamtrienones and an odor zone tentatively identified as TPB. Interestingly, both for TPB and megastigmatrienones, a remarkable variability associated with the vineyard site was observed, with sample Corvina A generating greater amounts of these compounds during aging. These compounds could therefore interesting markers in the selection of vineyards with greater capacity to provide wines expressing tobacco odor attributes during aging.

### Balsamic-like aroma compounds

**Figure 6:**
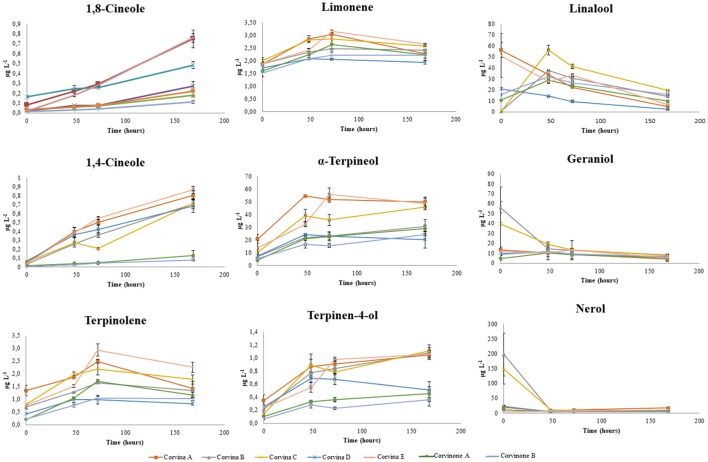
Evolution of terpenoids in Valpolicella wine samples during model aging.

The balsamic odor category contains resin, eucalyptus incense, resinous, and turpentine notes, with a variety of different aroma metabolites potentially contributing to these attributes. In this study, we focused on several terpenoids like cineoles and the terpinene series. Figure 6 shows the evolution of 1,4-cineole, reported to contribute to wine hay, dried herbs, and blackcurrant aromas (Antalick et al., 2015) and 1,8-cineole, indicated as potential contributors to eucalyptus and mint odors (Hervé et al., 2003). Both compounds increased in a linear manner during aging, indicating that in Corvina and Corvinone wines they could be considered as markers of aroma development. 1,8-Cineole has been previously observed in relatively high concentration in young Australian wines, while model wine studies indicated that its conversion rate from α-terpineol or limonene was rather low, around 0.6% (Capone et al., 2011). As this was considered too low to explain the high level found in young wines, the authors concluded that occurrence of 1,8-cineole in young Australian wines was due to airborne migration from eucalyptus tree to grapevine. A similar origin for 1,8-cineole was later proposed in relation to migration from other plants (Poitou et al., 2017). However, Fariña et al. (2005) proposed that 1,8-cineole could accumulate during wine aging through acid rearrangements of different terpenes, with limonene being the most relevant in Tannat wines. In the present study, 1,4- and 1,8-cineole were present in extremely low concentrations in young Valpolicella wines, but increased steadily during aging, in agreement with the observations of Fariña et al. (2005). It has to be pointed out that Corvina wines, and to a lesser extent Corvinone wines too, are relatively rich in free monoterpenes, although these are mostly linalool and α-terpineol, while limonene is present in lower concentrations (Supplementary Data 1). In order to gain further insights in the chemical pathways potentially leading to formation of the two cineoles, the evolution of different terpenes potentially involved in their formation was also studied during the wine aging experiment (Figure 6). Linalool, geraniol, β-citronellol in the first 48 h increased or decreased, presumably depending on availability of different precursors able to generate them during aging, in particular glycosidic precursors (Skouroumounis and Sefton, 2000) and terpene diols (Strauss et al., 1988). However, after this initial phase, these compounds decreased in all samples. In our data nerol showed, after an initial drastic decline, a slight increase at 168 h of storage. Linalool oxide showed an increase in the first 48 h of aging, but then the concentration was stable for the later 24 and 124 h of storage. Concerning cyclic monoterpenoids, α-terpineol and terpinen-4-ol generally tended to increase quickly in the first 48 h, and then to stabilize or slightly increase after 168 h. Conversely, terpinolene and limonene were observed to increase quite proportionally until 72 h of storage, and then clearly decrease after 168 h of model aging (Figure 6). It appeared therefore that during aging a complex equilibrium of hydratation/dehydratation acid catalyzed reactions involving α-terpineol, limonene, terpinen-4-ol, terpinolene, and other position isomers like α-terpinene and γ-terpinene took place. Additionally, formation of the cyclic terpenoid p-menthane-1,8-diol was also in some cases observed, as proposed by Rapp et al. (1985).

**Figure 7:**
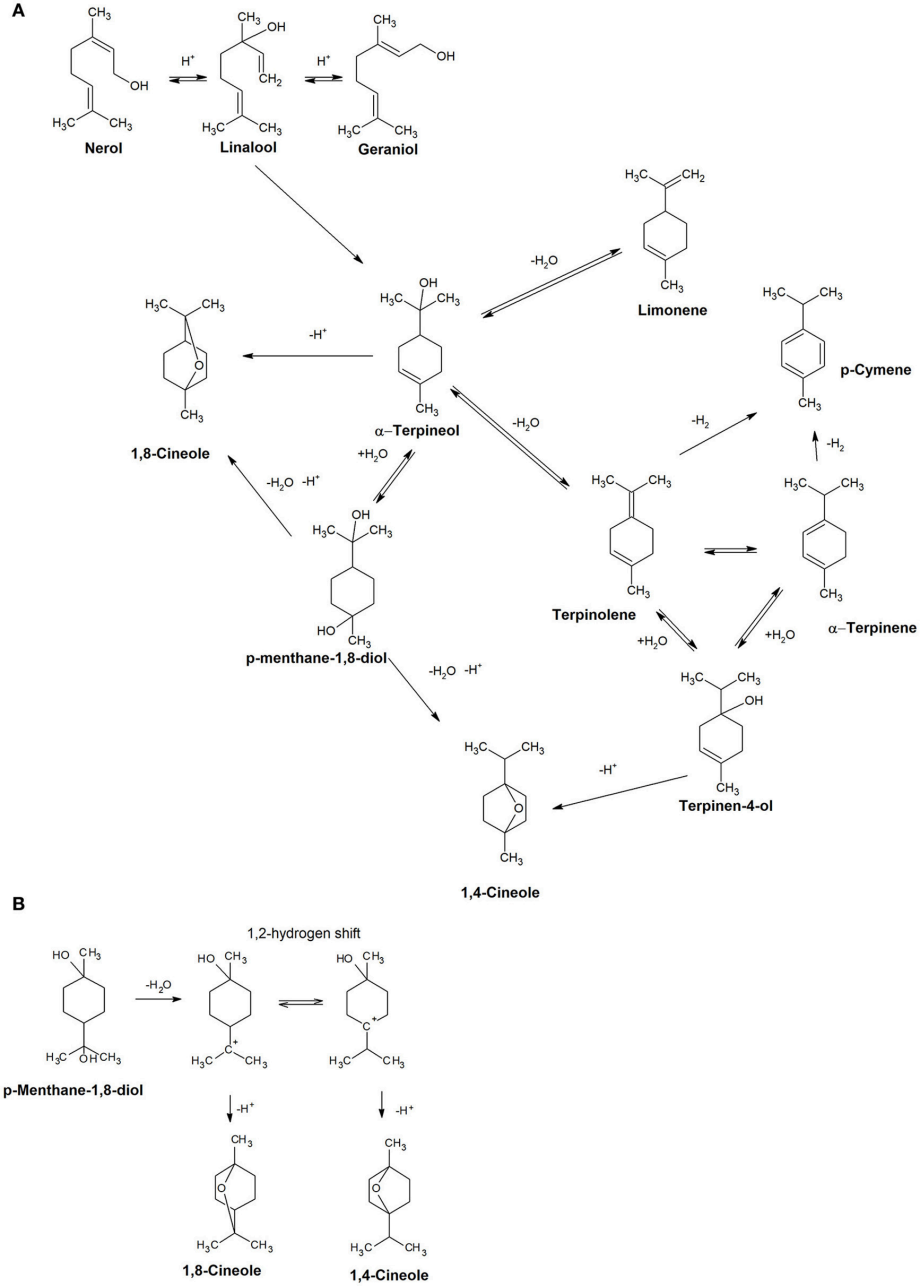
**(A)** Proposed acid catalyzed reactions involving terpenoids during aging. **(B)** Detail of the reaction occuring to p-menthane-1,8-diol to form 1,8-cineole or 1,4-cineole.

A model aging experiment was undertaken to rationalize the contribution of the various terpenes detected to cineoles formation, and the results are reported in Table 3. Addition of terpinen-4-ol in synthetic wine generated during aging 1,4-cineol but also, and more aboundantly, terpinolene, α-terpinene, γ-terpinene, terpinen-1-ol, and small amount of α-terpineol. α-Terpineol formed 1,8-cineol as described by Fariña et al. (2005), as well as terpinolene, α-terpinene, γ-terpinene, and limonene. When the same experiment was repeated using terpinolene and limonene as starting compounds, we observed the formation of α-terpinene, γ-terpinene, terpinen-1-ol, as well as more abundantly p-cymene,1,8-cineole, and 1,4-cineole. Obtained results made us propose the reactions scheme illustrated in Figure 7, supporting the view that that 1,8-cineole and 1,4-cineole are end products in the rearrangement reactions involving α-terpineol and terpinen-4-ol. Interestingly, when considering the period between 72 and 168 h of the model aging experiment involving real wines, it can be observed that the gain of concentration in 1,8-cineole was inversely correlated with the evolution of α-terpineol (*R*^2^ = 0.699), limonene (*R*^2^ = 0.722), and terpinolene (*R*^2^ = 0.641). Assuming that most of glycosidic precursors have already been hydrolysed before the 72 h, these data further confirming the contribution of these various free terpene compounds to 1,8-cineole formation during aging. Of these potential precursors, in the case of of Corvina and Corvinone α-terpineol appears to be quantitatively the most relevant.

**Table 3:** Compounds formed in model wine by single addition of precursors and stored at 60°C for 48, 72, and 168 h (nd: not detected; + trace; ++ medium level; + + + high level).

	**Reaction time (hours)**	**Compounds added to model wine**																	
		α**-terpineol**			**limonene**			**linalool**			**p-menthane-1,8-diol**			**terpinen-4-ol**			**terpinolene**		
		**48**	**72**	**160**	**48**	**72**	**160**	**48**	**72**	**160**	**48**	**72**	**160**	**48**	**72**	**160**	**48**	**72**	**160**
Formed compounds	1,8-cineole	++	++	++	+	+	+	nd	nd	+	nd	+	++	nd	nd	nd	+	+	+
	1,4-cineole	++	++	++	+	+	+	nd	nd	+	nd	+	++	+	+	++	+	+	+
	α-terpineol	+++	++	+	+++	++	+	+++	++	+	+	+	++	+	+	++	nd	nd	nd
	terpinen-1-ol	+	+	+	nd	nd	nd	nd	+	++	nd	+	++	+	+	++	nd	nd	nd
	terpinen-4-ol	+	+	+	+	nd	nd	+	+	+	nd	nd	+	+	+	nd	+	+	+
	terpinolene	++	+	+	+++	++	++	+++	++	+	+	+	++	nd	+	++	+	+	nd
	limonene	+	++	+++	+++	++	+	+++	++	+	+	+	++	nd	nd	nd	+	+	+
	α-terpinene	nd	nd	nd	nd	nd	nd	nd	nd	nd	nd	nd	nd	nd	+	++	nd	nd	nd
	γ-terpinene	+	++	+++	++	++	++	+	++	+++	+	+	++	nd	+	++	+	+	+
	p-cymene	+	++	+++	+++	++	+	+	++	+++	nd	nd	nd	+	++	+++	+++	++	+

**Figure 8:**
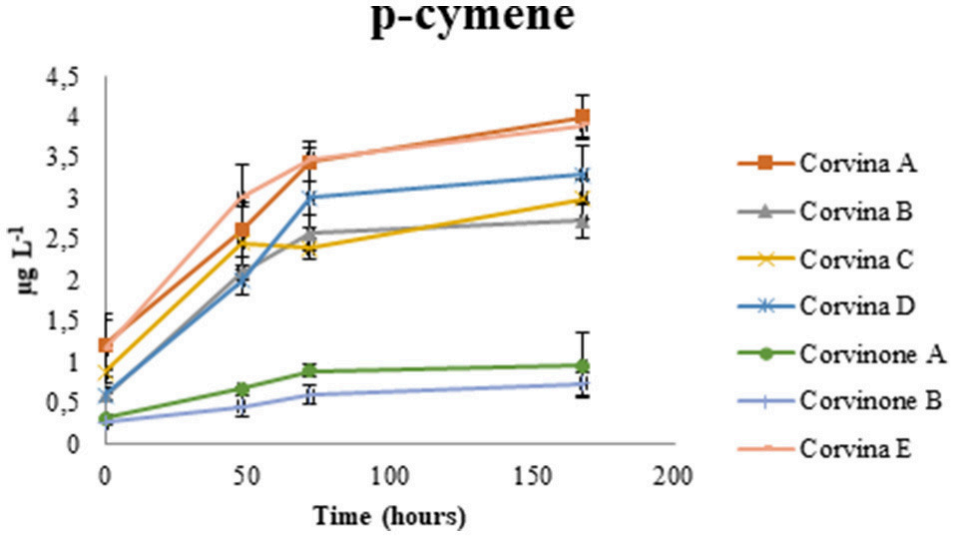
Accumulation of p-cymene in Valpolicella wines during model aging.

The cyclic terpenoid p-menthen-1,8-diol, previously reported by Rapp and Manderry (1986) as cyclization product of monoterpes, can also generate the two cineoles, presumably by dehydratation and direct cyclization or through formation of α-terpineol. In terms of compounds potentially contributing to balsamic odor notes of aged wines, during model aging we observed also a transient increase in the concentration of terpinolene, which has also been described as having a turpentine-like odor (Dvaranauskaite et al., 2008). In model wine spiked with terpinolene we observed formation of *p*-cymene, also described to have minty, solventy notes (Ruiz Pérez-Cacho and Rouseff, 2008) (Table 1), and this compound also increased during wine aging under our conditions (Figure 8).

Accumulation of 1,8-cineole varied substantially in relationship to grape origin, with the Corvina wine samples A and C exhibiting the highest content at the end of the aging experiment. Conversely, for 1,4-cineol and p-cymene a clear influence of the grape variety was observed, with Corvinone wines generating less of this compound during aging. The 2,10,10-trimethyl-6-methylene-1-oxa-spiro-[4.5]dec-7-ene, otherwise named vitispirane, has a camphoraceous aroma of chrysanthemum or earthy-woody undertone (Schulte-Elte et al., 1978). Data showed an increase of vitispirane during aging, in accordance with literature (Loscos et al., 2010), and it could participate to the balsamic bouquet of aged Corvina wines.

**Figure 9:**
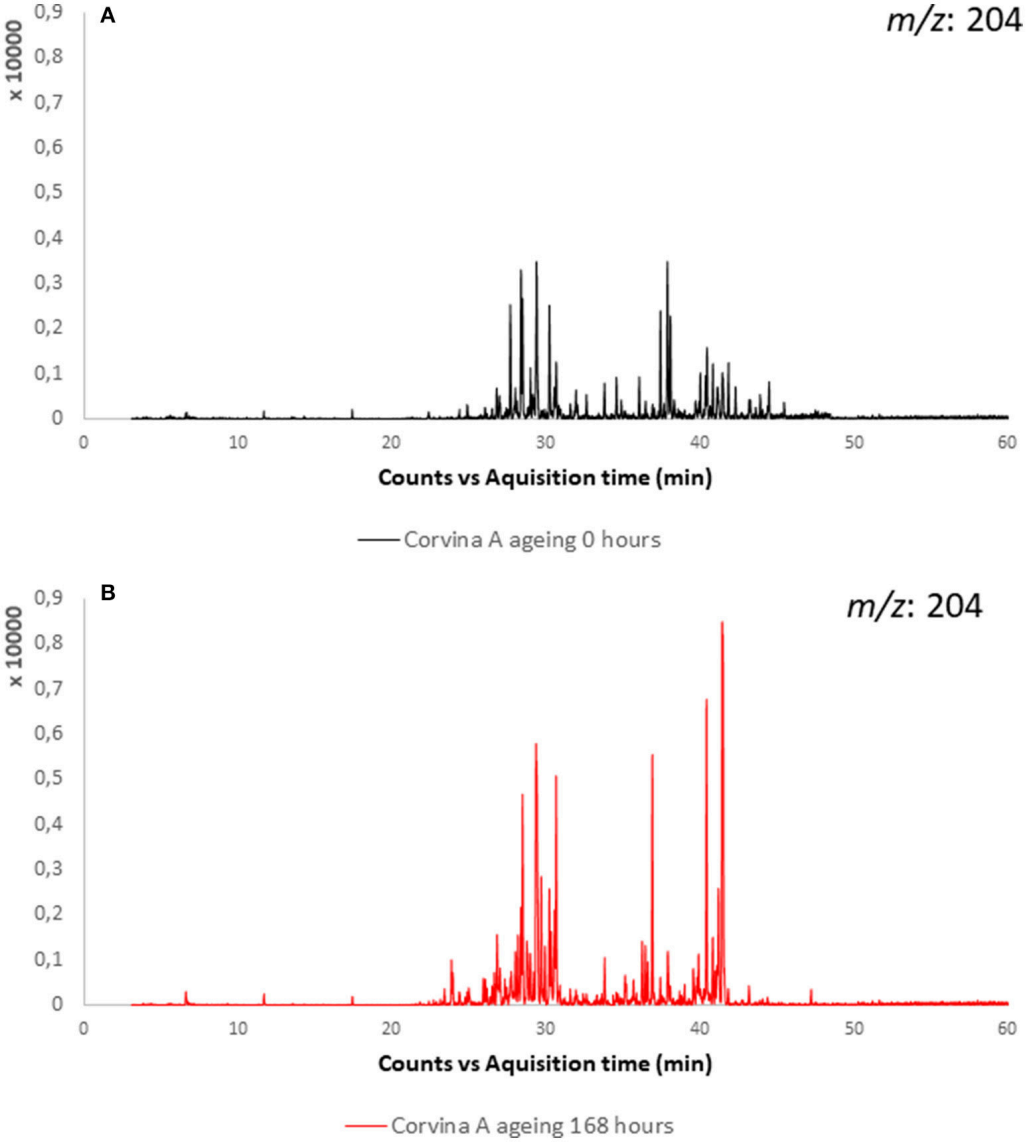
Extracted ion chromatogram (*m/z:* 204) of sesquiterpenes in Corvina A sample: **(A)** before aging; **(B)** and after 168 h of model aging.

Sesquiterpenoids are associated to peppery (Wood et al., 2008) or balsamic odor note (Zviely and Li, 2013). Little is known about the evolution of sesquiterpenes during wine aging. In the present study, the linear sesquiterpenes farnesol and nerolidol were detected in the experimental wines and their evolution was monitored during model aging, showing for both compounds a constant decrease during aging. Many additional peaks presenting the typical sesquiterpenes ions (204 *m/z* as molecular ion, and as recurring fragment ions the *m/z*: 189, 161, 93, 69) appeared and increased with model aging in studied wines (Figure 9). Those peaks were associated to sesquiterpenes considering also their fragmentation pattern (Enzell et al., 1984; Enzell and Wahlberg, 1986). Among these, on the basis of their mass spectra and comparison with NIST database (National Institute of Standards and Technology) (minimum of 80% of correspondence), two peaks were tentatively identified as α-chamigrene and bisabolol, respectively a cyclic and a bicyclic sesquiterpene. This last increased at 48 h of aging to decrease then after 72 and 160 h. Experiments conducted in model wine spiked with farnesol and nerolidol confirmed results obtained in wine, namely decrease of linear sesquiterpenes rearranging first in monocyclic structures like bisabolol and bisabolene, then to bicyclic sesquiterpenes with α-chamigrene-like structure. We suggest that, as in the case of linear terpenes (linalool, nerol, geraniol etc.), linear sesquiterpenes rearrange in acidic medium to form cyclic sesquiterpenes. Many other sesquiterpene peaks were formed during farnesol and nerolidol aging in model wine, but few of them were detected in Valpolicella wines. Furthermore, their identification was very difficult due to the low content, similar mass spectra. It was possible that different sesquiterpenes with very similar spectra may co-eluted.

Results showed in the present paper could be a starting point to further research on the behavior and role of sesquiterpenes in wine aging aroma.

## Conclusion

This paper provides a first chemical base to the observation that balsamic and tobacco aroma note appears during wine storage. The linear increase of compounds with tobacco like aroma, the TPB and the sum of megastigmatrienones isomers, during time has been proved. These molecules could be good marker of wine aging.

Concerning the balsamic aroma of aged Valpolicella wine, some terpenoids described in literature with minty, eucalyptus, camphoraceous, or turpentine-like odor, have been investigated.

In particular, a linear correlation has been found between wine aging and the bicyclic terpenoids 1,4-cineole, 1,8-cineole, and the cyclic terpene p-cymene. The formation of 1,4-cineole has been elucidated by proposing a formation pathway involving the terpinen-4-ol as starting point. In a similar manner, in Valpolicella wines the presence of 1,8-cineole has been attributed to the cyclization of α-terpineol occurring during wine storage, and not to an ambient contamination.

The 1,4-cineole and 1,8-cineole could be key compounds in the eucalyptus aroma of aged Valpolicella wines. The p-cymene has been suggested to come from precursors in the terpinene series. We suggested that the amount of 1,4-cineole and p-cymene was linked not only to wine aging but also to grape variety.

Sesquiterpenes have also been considered during analysis. We suggest that linear sesquiterpenes went through acid-catalyzed rearrangements leading to mono and bicyclic sesquiterpenes.

## Ethics statement

Our current work is only based on physicochemical analyses of commercial wines samples. It does not concern any sample issued from animal or human biological sources and, accordingly, does not require any ethics review process or written informed consents.

## Author contributions

DS performed experimental design and analysis. Data interpretation and article writing. MU performed experimental design, data interpretation and article writing, he is the project leader.

### Conflict of interest statement

The authors declare that the research was conducted in the absence of any commercial or financial relationships that could be construed as a potential conflict of interest.

## References

[B1] Antalick G. Perello M.-C. de Revel G. (2014). Esters in wines: new insight through the establishment of a database of french wines. Am. J. Enol. Vitic. 65, 293–304. 10.5344/ajev.2014.13133

[B2] Antalick G. Tempère S. Šuklje K. Blackman J. W. Deloire A. de Revel G.. (2015). Investigation and sensory characterization of 1,4-Cineole: a potential aromatic marker of Australian Cabernet Sauvignon Wine. J. Agric. Food Chem. 63, 9103–9111. 10.1021/acs.jafc.5b03847 26434979

[B3] Arbulu M. Sampedro M. C. Sanchez-Ortega A. Gómez-Caballero A. Unceta N. Goicolea M. A.. (2013). Characterisation of the flavour profile from Graciano Vitis vinifera wine variety by a novel dual stir bar sorptive extraction methodology coupled to thermal desorption and gas chromatography–mass spectrometry. Anal. Chim. Acta 777, 41–48. 10.1016/j.aca.2013.03.024 23622963

[B4] Baumes R. L. Aubert C. C. Günata Z. Y. De Moor W. Bayonove C. L. Tapiero C. (1994). Structures of two C13-norisoprenoid glucosidic precursors of wine flavour. J. Essent. Oil Res. 6, 587–599. 10.1080/10412905.1994.9699350

[B5] Bellincontro A. Matarese F. D'Onofrio C. Accordini D. Tosi E. Mencarelli F. (2016). Management of postharvest grape withering to optimise the aroma of the final wine: a case study on Amarone. Food Chem. 213, 378–387. 10.1016/j.foodchem.2016.06.098 27451194

[B6] Capone D. L. Van Leeuwen K. Taylor D. K. Jeffery D. W. Pardon K. H. Elsey G. M.. (2011). Evolution and occurrence of 1,8-cineole (Eucalyptol) in Australian wine. J. Agric. Food Chem. 59, 953–959. 10.1021/jf1038212 21204528

[B7] Cox A. Capone D. L. Elsey G. M. Perkins M. V. Sefton M. A. (2005). Quantitative analysis, occurrence, and stability of (E)-1-(2,3,6-Trimethylphenyl)buta-1,3-diene in wine. J. Agric. Food Chem. 53, 3584–3591. 10.1021/jf0479057 15853405

[B8] Díaz-Maroto M. C. Schneider R. Baumes R. (2005). Formation pathways of ethyl esters of branched short-chain fatty acids during wine aging. J. Agric. Food Chem. 53, 3503–3509. 10.1021/jf048157o 15853394

[B9] Dvaranauskaite A. Venskutonis P. R. Raynaud C. Talou T. Viškelis P. Dambrauskiene E. (2008). Characterization of steam volatiles in the essential oil of black currant buds and the antioxidant properties of different bud extracts. J. Agric. Food Chem. 56, 3279–3286 10.1021/jf7037168 18412360

[B10] Dziadas M. Jelen H. H. (2010). Analysis of terpenes in white wines using SPE–SPME–GC/MS approach. Anal. Chim. Acta 677, 43–49. 10.1016/j.aca.2010.06.035 20850588

[B11] Enzell C. R. Wahlberg I. (1986). Mass spectra of degraded tobacco isoprenoids. Mass Spec. Rev. 5, 39–72. 10.1002/mas.1280050103

[B12] Enzell C. R. Wahlberg I. Ryhage R. (1984). Mass spectra of tobacco isoprenoids. Mass Spect. Rev. 3, 395–438. 10.1002/mas.1280030304

[B13] Fariña L. Boido E. Carrau F. Versini G. Dellacassa E. (2005). Terpene compounds as possible precursors of 1,8-cineole in red grapes and wines. J. Agric. Food Chem. 53, 1633–1636. 10.1021/jf040332d 15740051

[B14] Fedrizzi B. Tosi E. Simonato B. Finato F. Cipriani M. Caramia G. (2011a). Changes in wine aroma composition according to botrytised berry percentage: a preliminary study on Amarone wine. Food Technol. Biotechnol. 49, 529–535.

[B15] Fedrizzi B. Zapparoli G. Finato F. Tosi E. Turri A. Azzolini M.. (2011b). Model aging and oxidation effects on varietal, fermentative, and sulfur compounds in a dry botrytized red wine. J. Agric. Food Chem. 59, 1804–1813. 10.1021/jf104160m 21314124

[B16] Francis I. L. Newton J. L. (2005). Determining wine aroma from compositional data. Aust. J. Grape Wine Res. 11, 114–126. 10.1111/j.1755-0238.2005.tb00283.x

[B17] Genovese A. Gambuti A. Piombino P. Moio L. (2007). Sensory properties and aroma compounds of sweet Fiano wine. Food Chem. 103, 1228–1236. 10.1016/j.foodchem.2006.10.027

[B18] Gocmen D. Gurbuz O. Rouseff R. L. Smoot J. M. Dagdelen A. F. (2004). Gas chromatographic-olfactometric characterization of aroma active compounds in sun-dried and vacuum-dried tarhana. Eur. Food Res. Technol. 218, 573–578. 10.1007/s00217-004-0913-6

[B19] Gunata Z. (2003). Flavor enhancement in fruit juices and derived beverages by exogenous glycosidases and consequences of the use of enzyme preparations, in Handbook of Food Enzymology, eds Whitaker J. R. Voragen A. G. J. Wong D. W. S. (New York, NY: Marcel Dekker Inc), 303–330.

[B20] Hervé E. Price S. Burns G. (2003). Eucalyptol in wines showing a “eucalyptus” aroma, in Proceedings of VIIeme Symposium International d'Oenologie, eds Lonvaud A. de Revel G. Darriet P. (Parise: Tec & Doc Lavoisier), 598–600.

[B21] Janusz A. Capone D. L. Puglisi C. J. Perkins M. V. Elsey G. M. Sefton M. A. (2003). (E)-1-(2,3,6-Trimethylphenyl)buta-1,3-diene: a potent grape-derived odorant in wine. J. Agric. Food Chem. 51, 7759–7763. 10.1021/jf0347113 14664541

[B22] Lorenzini M. Azzolini M. Tosi E. Zapparoli G. (2013). Postharvest grape infection of Botrytis cinerea and its interactions with other moulds under withering conditions to produce noble-rotten grapes. J. Appl. Microbiol. 114, 762–770. 10.1111/jam.12075 23163324

[B23] Loscos N. Hernández-Orte P. Cacho J. Ferreira V. (2010). Evolution of the aroma composition of wines supplemented with grape flavour precursors from different varietals during accelerated wine ageing. Food Chem. 120, 205–216. 10.1016/j.foodchem.2009.10.008

[B24] Makhotkina O. Kilmartin P. A. (2012). Hydrolysis and formation of volatile esters in New Zealand Sauvignon blanc wine. Food Chem. 135, 486–493. 10.1016/j.foodchem.2012.05.034 22868118

[B25] McKay M. Buglass A. J. Lee C. G. (2010). Wine, in Handbook of Alcoholic Beverages: Technical, Analytical and Nutritional Aspects, ed Buglass A. J. (Chichester: John Wiley & Sons, Ltd), 266–382.

[B26] Moio L. (2016). L'amarena e il pepe della Corvina, in Il Respiro del Vino, ed Moio L. (Milano: Mondadori), 459.

[B27] Picard M. Lytra G. Tempere S. Barbe J.-C. de Revel G. Marchand S. (2016). Identification of piperitone as an aroma compound contributing to the positive mint nuances perceived in aged red bordeaux wines. J. Agric. Food Chem. 64, 451–460. 10.1021/acs.jafc.5b04869 26735409

[B28] Poitou X. Thibon C. Darriet P. (2017). 1,8-Cineole in french red wines: evidence for a contribution related to its various origins. J. Agric. Food Chem. 65, 383–393. 10.1021/acs.jafc.6b03042 28060498

[B29] Rapp A. Güntert M. Ullemeyer H. (1985). Changes in aroma substances during the storage in bottles of white wines of the Riesling variety. Z. Lebensm. Unters. Forsch. 180, 109–116. 10.1007/BF01042633

[B30] Rapp A. Manderry H. (1986). Wine aroma. Experentia 42, 857–966. 10.1007/BF01941764

[B31] Ribéreau-Gayon P. Glories Y. Maujean A. Dubourdieu D. (2006). Varietal aroma, in Handbook of Enology, The Chemistry of Wine: Stabilization and Treatments, 2nd Edn. (Chichester, UK: John Wiley & Sons Ltd), 213.

[B32] Rolle L. Giacosa S. Río Segade S. Ferrarini R. Torchio F. Gerbi V. (2013). Influence of different thermohygrometric conditions on changes in instrumental texture properties and phenolic composition during postharvest withering of “corvina” winegrapes (*Vitis vinifera L*.). Drying Technol. 31, 549–564. 10.1080/07373937.2012.745092

[B33] Ruiz Pérez-Cacho P. Rouseff R. (2008). Processing and storage effects on orange juice aroma: a review. J. Agric. Food Chem. 56, 9785–9796. 10.1021/jf801244j 18828595

[B34] Schreier P. Drawert F. (1974). Investigation of volatile components in wine by gaschromatography and mass- spectrometry. I. Nonpolar Compounds Wine-Flavour. Lebensm. Unters Forsch. 154, 273–278. 10.1007/BF01083422

[B35] Schulte-Elte K. H. Gautschi F. Renold W. Hauser A. Fankhauser P. Limacher J. (1978). Vitispiranes, important constituents of vanilla aroma. Helv. Chim. Acta 61, 1125–1133. 10.1002/hlca.19780610326

[B36] Silva Ferreira A. C. Hogg T. Guedes De Pinho P. (2003). Identification of key odorants related to the typical aroma of oxidation-spoiled white wines. J. Agric. Food Chem. 51, 1377–1381. 10.1021/jf025847o 12590484

[B37] Simpson R. F. Strauss C. R. Williams P. J. (1977). Vitispirane C-13 spiro-ether in aroma volatiles of grape juice, wines and distilled grape spirits. Chem. Ind. 15, 663–664.

[B38] Skouroumounis G. K. Massy-Westropp R. A. Sefton M. A. Williams P. J. (1992). Precursors of damascenone in fruit juices. Tetrahedron Lett. 33, 3533–3536. 10.1016/S0040-4039(00)92682-0

[B39] Skouroumounis G. K. Sefton M. A. (2000). Acid-catalyzed hydrolysis of alcohols and their β-D-glucopyranosides. J. Agric. Food Chem. 48, 2033–2039. 10.1021/jf9904970 10888494

[B40] Slaghenaufi D. Marchand-Marion S. Richard T. Waffo-Teguo P. Bisson J. Monti J.-P.. (2013). Centrifugal partition chromatography applied to the isolation of oak wood aroma precursors. Food Chem. 141, 2238–2245. 10.1016/j.foodchem.2013.04.069 23870953

[B41] Slaghenaufi D. Perello M.-C. Marchand-Marion S. de Revel G. (2014). Quantitative solid phase microextraction–gas chromatography mass spectrometry analysis of five megastigmatrienone isomers in aged wine. Anal. Chim. Acta 813, 63–69. 10.1016/j.aca.2014.01.019 24528661

[B42] Slaghenaufi D. Perello M.-C. Marchand-Marion S. de Revel G. (2016). Quantification of megastigmatrienone, a potential contributor to tobacco aroma in spirits. Food Chem. 203, 41–48. 10.1016/j.foodchem.2016.02.034 26948587

[B43] Stefanini I. Carlin S. Tocci N. Albanese D. Donati C. Franceschi P.. (2017). Core microbiota and metabolome of *Vitis vinifera L*. cv. Corvina grapes and musts. Front. Microbiol. 8:457. 10.3389/fmicb.2017.00457 28377754 PMC5359246

[B44] Strauss C. R. Wilson B. Williams P. J. (1987). 3-oxo-α-ionol, vomifoliol and roseoside in Vitis vinifera fruit. Phytochemistry 26, 1995–1997.

[B45] Strauss C. R. Wilson B. Williams P. J. (1988). Novel Monoterpene Diols and Diol Glycosides in Vitis vinifera Grapes. J. Agric. Food Chem. 36, 569–573. 10.1021/jf00081a041

[B46] Tatum J. Nagy S. Berry R. (1975). Degradation products formed in canned single-strength orange juice during storage. J. Food Sci. 40, 707–709. 10.1111/j.1365-2621.1975.tb00536.x

[B47] Ugliano M. Kolouchova R. Henschke P. A. (2011). Occurrence of hydrogen sulfide in wine and in fermentation: influence of yeast strain and supplementation of yeast available nitrogen. J. Ind. Microbiol. Biotechnol. 38, 423–429. 10.1007/s10295-010-0786-6 20668912

[B48] van Den Dool H. Kratz P. D. (1963). A generalization of the retention index system including linear temperature programmed gas-liquid partition chromatography. J. Chromatogr. 11, 463–471. 10.1016/S0021-9673(01)80947-X 14062605

[B49] Wedler H. B. Pemberton R. P. Tantillo D. J. (2015). Carbocations and the complex flavor and bouquet of wine: mechanistic aspects of terpene biosynthesis in wine grapes. Molecules 20, 10781–10792. 10.3390/molecules200610781 26111168 PMC6272345

[B50] Weyerstahl P. Meisel T. Mewes K. Negahdari S. (1991). Struktur und Geruch, XIII. Synthese und olfaktorische Eigenschaften von Megastigmatrienon-Analoga. Liebigs Annalen der Chemie 1, 19–25. 10.1002/jlac.199119910104

[B51] Winterhalter P. (1991). 1,1,6-Trimethyl-1,2-dihydronaphthalene (TDN) formation in wine. 1. Studies on the hydrolysis of 2,6,10,10-Tetramethyl-1-oxaspiro[4.5]dec- 6-ene-2,8-diol rationalizing the origin of TDN and related C13 norisoprenoids in riesling wine. J. Agric. Food Chem. 39, 1825–1829. 10.1021/jf00010a027

[B52] Winterhalter P. Skouroumounis G. K. (1997). Glycoconjugated aroma compounds: occurrence, role and biotechnological transformation, in Advances in Biochemical Engineering/Biotechnology, ed Scheper T. (Heidelberg; Berlin: Spring-Verlag), 74–105. 10.1007/BFb0102063 9017925

[B53] Wood C. Siebert T. E. Parker M. D. L. Capone G. M. Elsey A. P. Pollnitz A. P. (2008). From wine to pepper: rotundone, an obscure sesquiterpene, is a potent spicy aroma compound. J. Agric. Food Chem. 10, 3738–3744. 10.1021/jf800183k 18461961

[B54] Zamboni A. Minoia L. Ferrarini A. Tornielli G. B. Zago E. Delledonne Pezzotti M. (2008). Molecular analysis of post-harvest withering in grape by AFLP transcriptional profiling. J. Exp. Bot. 59, 4145–4159. 10.1093/jxb/ern256 19010774 PMC2639028

[B55] Zoccatelli G. Zenoni S. Savoi S. Dal Santo S. Tononi P. Zandonà V. (2013). Skin pectin metabolism during the postharvest dehydration of berries from three distinct grapevine cultivars. Aust. J. Grape Wine Res. 19, 171–179. 10.1111/ajgw.12014

[B56] Zviely M. Li M. (2013). Sesquiterpenoids: the Holy Fragrance Ingredients. Perfumer Flavorist 38, 52–55.

