# Supplementary material for: Norisoprenoids, Sesquiterpenes and Terpenoids Content of Valpolicella Wines During Aging: Investigating Aroma Potential in Relationship to Evolution of Tobacco and Balsamic Aroma in Aged Wine
Source: Front Chem. 2018 Mar 19;6:66. doi: 10.3389/fchem.2018.00066 (PMC5867301; doi:10.3389/fchem.2018.00066)
Supplement: Supplementary file 1 [file DataSheet1.DOCX]

**Supplementary data 1**. Compounds concentration in µg L^-1^.

| **Sample** | **Corvina A** | | | | **Corvina B** | | | | **Corvina C** | | | | **Corvina D** | | | | **Corvina E** | | | | **Corvinone A** | | | | **Corvinone B** | | | |
| --- | --- | --- | --- | --- | --- | --- | --- | --- | --- | --- | --- | --- | --- | --- | --- | --- | --- | --- | --- | --- | --- | --- | --- | --- | --- | --- | --- | --- |
| **Ageing time (hours)** | **0** | **48** | **72** | **168** | **0** | **48** | **72** | **168** | **0** | **48** | **72** | **168** | **0** | **48** | **72** | **168** | **0** | **48** | **72** | **168** | **0** | **48** | **72** | **168** | **0** | **48** | **72** | **168** |
| **γ-Terpinene** | 2,83 | 0,75 | 12,18 | 11,36 | 1,28 | 9,62 | 15,37 | 12,81 | 1,90 | 11,85 | 9,43 | 10,11 | 1,52 | 7,76 | 7,63 | 6,00 | 1,62 | 11,88 | 11,53 | 12,14 | 0,33 | 2,09 | 3,73 | 6,44 | 0,53 | 1,69 | 2,87 | 3,28 |
| **Terpinen-1-ol** | 0,01 | 0,16 | 0,20 | 0,39 | <LOD | 0,07 | 0,01 | 0,33 | <LOD | 0,01 | 0,02 | 0,40 | 0,01 | <LOD | 0,21 | 0,31 | 0,01 | 0,24 | 0,01 | 0,39 | <LOD | <LOD | 0,01 | 0,03 | 0,01 | 0,01 | 0,01 | 0,01 |
| **p-Cymene** | 1,20 | 2,61 | 3,45 | 4,00 | 0,58 | 2,09 | 2,57 | 2,72 | 0,87 | 2,45 | 2,39 | 2,99 | 0,60 | 2,00 | 3,01 | 3,29 | 1,16 | 3,00 | 3,48 | 3,89 | 0,32 | 0,66 | 0,89 | 0,96 | 0,26 | 0,46 | 0,60 | 0,73 |
| **1,4-Cineole** | 0,05 | 0,40 | 0,50 | 0,80 | 0,03 | 0,26 | 0,36 | 0,71 | 0,05 | 0,27 | 0,21 | 0,71 | 0,07 | 0,36 | 0,42 | 0,68 | 0,04 | 0,39 | 0,55 | 0,87 | 0,01 | 0,04 | 0,05 | 0,13 | <LOD | 0,03 | 0,04 | 0,08 |
| **Bisabolol** | 0,48 | 2,46 | 2,38 | 1,48 | 0,22 | 1,24 | 1,75 | 2,83 | 0,88 | 3,76 | 4,03 | 5,57 | 1,10 | 5,99 | 5,06 | 2,55 | 0,69 | 10,77 | 10,50 | 14,41 | 0,38 | 2,37 | 3,05 | 3,30 | 0,30 | 1,75 | 2,20 | 3,30 |
| **1,8-Cineole** | 0,08 | 0,22 | 0,29 | 0,75 | 0,03 | 0,06 | 0,07 | 0,18 | 0,02 | 0,07 | 0,07 | 0,27 | 0,16 | 0,25 | 0,26 | 0,48 | 0,02 | 0,18 | 0,28 | 0,76 | 0,04 | 0,06 | 0,08 | 0,22 | 0,01 | 0,03 | 0,04 | 0,11 |
| **Limonene** | 1,86 | 2,84 | 3,03 | 2,27 | 1,87 | 2,33 | 2,46 | 2,42 | 2,00 | 2,81 | 2,85 | 2,58 | 1,71 | 2,07 | 2,06 | 1,93 | 1,87 | 2,41 | 3,15 | 2,65 | 1,59 | 2,23 | 2,63 | 2,24 | 1,52 | 2,08 | 2,22 | 2,22 |
| **Terpinolene** | 1,35 | 1,87 | 2,50 | 1,42 | 0,70 | 1,29 | 1,65 | 1,35 | 0,80 | 1,97 | 2,20 | 1,78 | 0,43 | 0,98 | 0,99 | 0,84 | 0,76 | 1,51 | 2,94 | 2,26 | 0,21 | 1,05 | 1,71 | 1,17 | 0,22 | 0,76 | 1,04 | 1,02 |
| **cis-Linalooloxide** | 0,65 | 3,97 | 6,76 | 0,20 | 0,11 | 1,34 | 5,54 | 1,65 | 0,07 | 2,30 | 5,99 | 5,19 | 0,26 | 5,67 | 1,44 | 1,04 | 0,64 | 3,35 | 5,39 | 0,20 | 0,16 | 7,07 | 8,39 | 6,93 | 0,17 | 1,05 | 5,88 | 1,49 |
| **trans-Linalooloxide** | <LOD | 0,02 | 0,03 | 0,01 | 0,04 | 0,01 | 0,02 | 0,01 | 0,02 | 0,01 | 0,03 | 0,02 | <LOD | 0,01 | 0,01 | 0,04 | <LOD | 0,03 | 0,04 | 0,02 | <LOD | 0,01 | 0,02 | 0,05 | <LOD | 0,01 | 0,01 | 0,01 |
| **sesquiterpenenes 1^a^** | 0,16 | 0,23 | 0,58 | 0,31 | 17,99 | 3,11 | 4,08 | 2,85 | 6,42 | 2,16 | 2,79 | 1,51 | 2,44 | 5,96 | 5,08 | 2,50 | 0,67 | 1,97 | 3,51 | 2,18 | 0,01 | 0,50 | 0,65 | 0,37 | 0,01 | 0,39 | 0,02 | 0,47 |
| **Linalool** | 56,24 | 33,89 | 22,45 | 4,96 | 0,40 | 37,43 | 30,44 | 14,03 | 0,33 | 56,16 | 41,22 | 19,49 | 21,14 | 14,39 | 9,56 | 2,47 | 51,21 | 27,38 | 33,34 | 6,51 | 10,71 | 28,94 | 23,87 | 9,80 | 15,69 | 32,02 | 26,49 | 15,87 |
| **Terpinen-4-ol** | 0,35 | 0,87 | 0,91 | 1,05 | 0,21 | 0,77 | 0,84 | 1,08 | 0,14 | 0,89 | 0,78 | 1,12 | 0,26 | 0,69 | 0,68 | 0,51 | 0,24 | 0,55 | 0,98 | 1,06 | 0,11 | 0,33 | 0,36 | 0,45 | 0,06 | 0,29 | 0,23 | 0,36 |
| **sesquiterpene 2 ^a^** | 0,09 | 0,07 | 0,43 | 0,42 | 0,28 | 2,47 | 3,41 | 2,92 | 0,29 | 1,67 | 2,32 | 1,66 | 0,30 | 6,63 | 6,37 | 6,73 | 0,67 | 1,96 | 3,29 | 4,41 | 0,02 | 0,24 | 0,34 | 0,36 | 0,01 | 0,12 | 0,13 | 0,25 |
| **sesquiterpene 4 ^a^** | 0,03 | 0,06 | 0,17 | 0,17 | 0,30 | 0,51 | 0,85 | 0,90 | 0,09 | 0,55 | 0,95 | 0,81 | 0,66 | 4,39 | 4,38 | 4,63 | 0,18 | 0,52 | 0,88 | 1,21 | <LOD | 0,31 | 0,51 | 0,48 | 0,01 | 0,16 | 0,08 | 0,32 |
| **Sesquiterpene 5 ^a^** | 0,10 | 0,39 | 1,78 | 1,20 | 0,40 | 3,37 | 5,31 | 5,63 | 0,38 | 3,00 | 4,50 | 4,11 | 0,63 | 12,68 | 13,56 | 12,26 | 0,84 | 5,05 | 21,26 | 13,94 | 0,04 | 1,68 | 2,41 | 2,37 | <LOD | 1,06 | 0,66 | 2,08 |
| **sesquiterpene 6 ^a^** | 0,13 | 0,31 | 0,52 | 0,43 | 0,17 | 1,06 | 1,62 | 1,66 | 0,22 | 0,99 | 1,43 | 1,01 | 0,93 | 3,61 | 3,37 | 3,52 | 0,69 | 1,20 | 3,45 | 3,18 | 0,08 | 0,64 | 1,08 | 0,84 | 0,27 | 0,44 | 0,64 | 0,85 |
| **α-Terpineol** | 20,87 | 54,74 | 51,81 | 50,02 | 7,02 | 21,86 | 23,29 | 30,81 | 10,34 | 39,25 | 35,80 | 45,94 | 7,61 | 24,35 | 22,99 | 20,43 | 13,90 | 32,29 | 55,97 | 48,82 | 4,21 | 21,35 | 22,94 | 29,17 | 5,39 | 16,62 | 15,72 | 24,23 |
| **TPB ^a^** | 0,01 | 2,24 | 2,97 | 8,57 | 0,06 | 1,08 | 1,60 | 4,08 | 0,11 | 1,63 | 2,35 | 6,82 | <LOD | 1,95 | 2,18 | 5,95 | 0,56 | 0,76 | 2,54 | 5,99 | <LOD | 1,08 | 1,71 | 5,35 | 0,05 | 0,83 | 1,03 | 3,79 |
| **TDN ^a^** | 4,79 | 225,36 | 363,64 | 1008,75 | 4,13 | 63,98 | 113,55 | 367,77 | 1,84 | 82,97 | 135,41 | 415,64 | 4,47 | 178,59 | 244,30 | 745,57 | 11,57 | 128,26 | 331,31 | 1123,83 | 1,67 | 121,90 | 244,01 | 779,57 | 2,28 | 48,33 | 86,96 | 331,15 |
| **Sesquiterpene 7 ^a^** | 1,36 | 1,02 | 0,82 | 0,64 | 53,76 | 5,15 | 1,26 | 1,24 | 51,62 | 2,19 | 1,72 | 5,57 | 1,48 | 2,83 | 2,19 | 2,33 | 2,16 | 1,47 | 3,32 | 1,54 | 0,57 | 1,79 | 1,60 | 0,66 | 1,06 | 6,04 | 0,93 | 5,38 |
| **β-Citronellol** | 11,77 | 6,95 | 5,39 | 0,27 | 0,83 | 20,25 | 17,43 | 10,28 | 0,79 | 23,85 | 17,56 | 11,13 | 15,55 | 8,35 | 8,14 | 0,61 | 13,68 | 6,29 | 1,72 | 0,99 | 6,99 | 11,64 | 10,19 | 5,41 | 14,50 | 15,08 | 11,20 | 9,16 |
| **Nerol** | 20,00 | 6,73 | 10,90 | 18,31 | 197,69 | 8,44 | 9,30 | 8,67 | 148,26 | 11,20 | 10,80 | 9,60 | 23,59 | 5,56 | 5,71 | 8,46 | 5,47 | 8,61 | 8,62 | 12,37 | 12,00 | 6,65 | 7,86 | 7,77 | 4,26 | 6,02 | 4,36 | 5,30 |
| **β-Damascenone** | 6,35 | 7,05 | 6,44 | 5,87 | 5,46 | 5,70 | 5,07 | 4,28 | 0,03 | 6,29 | 5,14 | 4,66 | 3,13 | 4,31 | 3,66 | 3,44 | 5,40 | 4,12 | 6,62 | 5,32 | 2,43 | 5,89 | 5,78 | 5,49 | 4,90 | 6,30 | 5,56 | 4,79 |
| **Geraniol** | 13,51 | 10,27 | 8,57 | 4,13 | 56,32 | 14,34 | 12,67 | 8,15 | 39,84 | 18,82 | 13,59 | 7,98 | 10,02 | 10,96 | 8,91 | 6,11 | 11,95 | 10,41 | 13,54 | 5,30 | 4,83 | 10,14 | 8,79 | 5,29 | 8,86 | 11,64 | 9,10 | 6,80 |
| **Nerolidol (sum of isomers)** | 0,93 | 0,91 | 0,80 | 0,12 | 62,52 | 2,05 | 1,90 | 0,78 | 66,82 | 4,94 | 3,95 | 1,31 | 2,46 | 2,55 | 1,41 | 0,21 | 1,98 | 4,00 | 15,23 | 1,16 | 0,68 | 3,01 | 2,69 | 0,81 | 1,02 | 3,00 | 3,13 | 1,65 |
| **p-Menthane-1,8-diol** | <LOD | <LOD | <LOD | <LOD | <LOD | <LOD | <LOD | <LOD | <LOD | <LOD | <LOD | <LOD | <LOD | <LOD | <LOD | <LOD | <LOD | <LOD | <LOD | <LOD | <LOD | <LOD | <LOD | <LOD | <LOD | <LOD | <LOD | <LOD |
| **8-Hydroxylinalool ^a^** | <LOD | <LOD | <LOD | <LOD | <LOD | <LOD | <LOD | <LOD | <LOD | <LOD | <LOD | <LOD | <LOD | <LOD | <LOD | <LOD | <LOD | <LOD | <LOD | <LOD | <LOD | <LOD | <LOD | <LOD | <LOD | <LOD | <LOD | <LOD |
| **Farnesol (sum of isomers)** | 2,50 | 0,52 | 0,38 | 0,15 | 4,38 | 1,91 | 1,34 | 0,32 | 5,96 | 5,07 | 2,72 | 0,53 | 5,11 | 1,35 | 0,68 | 0,11 | 12,68 | 2,33 | 7,56 | 0,31 | 2,96 | 2,36 | 1,46 | 0,43 | 5,13 | 3,46 | 2,28 | 0,73 |
| **3-Oxo-α-ionol ^a^** | 0,01 | 0,01 | 0,06 | 0,04 | 0,01 | 0,05 | 0,04 | 0,05 | 0,01 | 0,05 | 0,05 | 0,05 | <LOD | 0,07 | 0,05 | 0,10 | 0,01 | <LOD | 0,15 | 0,02 | 0,01 | 0,08 | 0,06 | 0,05 | <LOD | 0,05 | 0,12 | 0,04 |
| **Vitispirane 1 ^a^** | 8,1 | 178 | 254 | 480 | 0,1 | 59 | 96,3 | 227 | 4,8 | 128 | 58,4 | 280 | 4,8 | 166 | 188 | 349 | 4,4 | 109 | 204 | 410 | 2,5 | 143 | 257 | 601 | 2,3 | 57,6 | 101 | 309 |
| **Vitispirane 2 ^a^** | 8,1 | 106 | 254 | 480 | 1,6 | 39,4 | 63 | 227 | 4,8 | 128 | 37,2 | 145 | 4,8 | 166 | 97,2 | 178 | 2,4 | 50,9 | 204 | 410 | 2,5 | 143 | 238 | 601 | 3,1 | 65,8 | 105 | 257 |
| **Megastigmatrienone (sum of isomers)** | 0,68 | 2,03 | 9,09 | 20,79 | 0,81 | 4,69 | 4,10 | 8,33 | 0,52 | 4,11 | 4,47 | 9,97 | 1,45 | 7,07 | 7,88 | 12,22 | 0,98 | 3,22 | 7,50 | 12,22 | 0,31 | 4,25 | 4,53 | 11,41 | 1,18 | 3,36 | 0,95 | 6,55 |

^a^ quantified as µg L^-1^ of octan-2-ol.
